# Bis(2,2′-bipyridine-κ^2^ *N*,*N*′)bis­[3-(3-hy­droxyphenyl)propenoato-κ^2^ *O* ^1^,*O* ^1′^]cadmium

**DOI:** 10.1107/S1600536811050847

**Published:** 2011-11-30

**Authors:** Chun-Yan Zhang, Yi-Hang Wen

**Affiliations:** a Zhejiang Key Laboratory for Reactive Chemistry on Solid Surfaces, Institute of Physical Chemistry, Zhejiang Normal University, Jinhua, Zhejiang 321004, People’s Republic of China

## Abstract

The title compound, [Cd(C_9_H_7_O_3_)_2_(C_10_H_8_N_2_)_2_], was synthesized under mild hydro­thermal conditions. The structure of the complex mol­ecule consists of four approximately planar fragments: two 3-(3-hy­droxyphenyl)propenoate residues and two 2,2′-bipyridine ligands [largest deviation from the least-squares planes is 0.240 (1) Å for one of 3-(3-hy­droxy­phenyl)propenoate residues]. The dihedral angles formed by the least-squares planes of the 2,2′-bipyridine ligands and the opposite 3-(3-hy­droxyphenyl)propenoate residues are 22.68 (7) and 26.47 (6)°. The CdN_4_O_4_ coordination polyhedron can be described as distorted dodecahedral. Inter­molecular O—H⋯O hydrogen bonds between carboxyl­ate O atoms and hy­droxy groups lead to the formation of chains along the *a*-axis direction.

## Related literature

For carboxyl­ate complexes, see: Armentano *et al.* (2005 ▶); Baca *et al.* (2005 ▶); Karmakar *et al.* (2009 ▶); Liu *et al.* (2008 ▶); Rao *et al.* (2004 ▶); Zhao *et al.* (2010 ▶); Zhu *et al.* (2011 ▶).
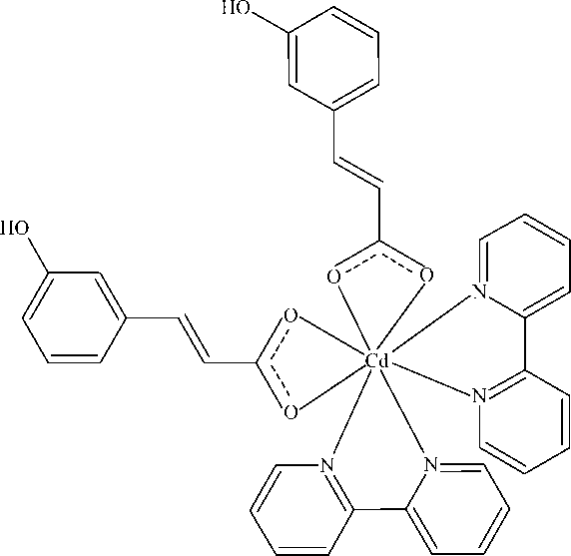


## Experimental

### 

#### Crystal data

[Cd(C_9_H_7_O_3_)_2_(C_10_H_8_N_2_)_2_]
*M* *_r_* = 751.06
Monoclinic, 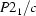

*a* = 8.7769 (16) Å
*b* = 36.711 (7) Å
*c* = 13.188 (2) Å
β = 126.382 (10)°
*V* = 3421.0 (10) Å^3^
*Z* = 4
Mo *K*α radiation
μ = 0.69 mm^−1^
*T* = 296 K
0.35 × 0.25 × 0.10 mm

#### Data collection

Bruker APEXII CCD area-detector diffractometer
Absorption correction: multi-scan (*SADABS*; Bruker, 2009 ▶) *T*_min_ = 0.81, *T*_max_ = 0.93
29901 measured reflections
7869 independent reflections
6136 reflections with *I* > 2σ(*I*)
*R*_int_ = 0.034

#### Refinement

*R*[*F* ^2^ > 2σ(*F* ^2^)] = 0.036
*wR*(*F* ^2^) = 0.076
*S* = 1.02
7869 reflections
442 parameters
1 restraint
H-atom parameters constrained
Δρ_max_ = 0.48 e Å^−3^
Δρ_min_ = −0.53 e Å^−3^

### 

Data collection: *APEX2* (Bruker, 2009 ▶); cell refinement: *SAINT* (Bruker, 2009 ▶); data reduction: *SAINT*; program(s) used to solve structure: *SHELXS97* (Sheldrick, 2008 ▶); program(s) used to refine structure: *SHELXL97* (Sheldrick, 2008 ▶); molecular graphics: *SHELXTL* (Sheldrick, 2008 ▶); software used to prepare material for publication: *SHELXTL*.

## Supplementary Material

Crystal structure: contains datablock(s) I, global. DOI: 10.1107/S1600536811050847/yk2032sup1.cif

Structure factors: contains datablock(s) I. DOI: 10.1107/S1600536811050847/yk2032Isup2.hkl

Additional supplementary materials:  crystallographic information; 3D view; checkCIF report

## Figures and Tables

**Table 1:** Hydrogen-bond geometry (Å, °)

*D*—H⋯*A*	*D*—H	H⋯*A*	*D*⋯*A*	*D*—H⋯*A*
O6—H6*B*⋯O5^i^	0.82	1.86	2.672 (3)	171
O3—H3*B*⋯O1^ii^	0.82	1.82	2.624 (3)	168

Symmetry codes: (i) 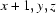; (ii) 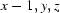.

**Fig. 1.**
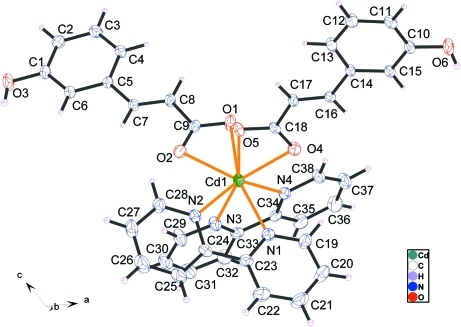
The molecular structure of the title compound, showing 30% displacement ellipsoids for non-H atoms and atom labelling scheme.

**Fig. 2.**
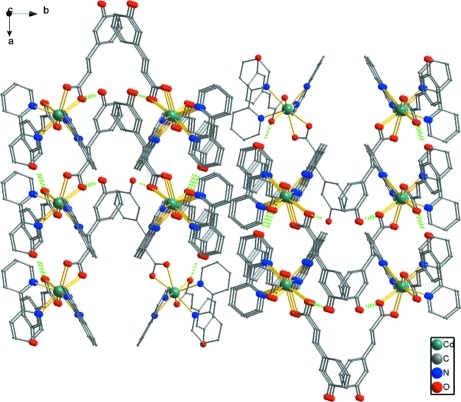
Molecular packing diagram of the title compound showing the hydrogen bonding interactions.

## supplementary crystallographic information

### Comment

Carboxylate complexes have received a great deal of attention over the last few years due to their interesting coordination chemistry, unusual structural features, remarkable physical and chemical properties, and extensive practical applications (Baca *et al.*, 2005; Rao *et al.*, 2004). Very recently, the coordination chemistry of carboxylate groups with transition metals, especially importing the ancillary ligand (*e.g.* pyridine), has undergone extensive development. (Armentano *et al.*, 2005; Karmakar *et al.*, 2009; Zhu *et al.*,2011; Zhao *et al.*,2010; Liu *et al.*,2008). In this context, we report the synthesis and structure of a new compound, [Cd(C_9_H_7_O_3_)_2_(C_10_H_8_N_2_)_2_].

A perspective view of molecular structure of the title complex is presented in Fig.1. The Cd^2+^ ions are eight-coordinated by two carboxylate groups from two 3-(3-hydroxyphenyl)propenoate anions, and four nitrogen atoms from two 2,2'-bipyridine molecules. The structure of complex molecule consists of four approximately planar fragments: two 3-(3-hydroxyphenyl)propenoate residues and two 2,2'-bipyridine ligands (largest deviation from the l.s. planes is 0.240 (1) Å for one of 3-(3-hydroxyphenyl)propenoate residues). The dihedral angles formed by the l.s. planes of 2,2'-bipyridine ligands and opposite 3-(3-hydroxyphenyl)propenoate residues are 22.68 (7)° and 26.47 (6)°. Strong intermolecular hydrogen bonding interactions O—H···O (2.672 (3) Å and 2.624 (3) Å) between the carboxylate O atoms and hydroxy groups play a vital role in the construction of the chains along the *a* direction (Figure. 2).

### Experimental

A mixture of Cd(Ac)_2_.6H_2_O (0.1998 g, 0.5 mmol), 3-hydroxycinnamic acid (0.1642 g, 1 mmol)and 2,2'-bipyridine (0.1563 g, 1 mmol) was dissolved in 25 ml of EtOH/ H_2_O mixture (V/V,1:5), and then sealed in a 25 ml Teflon-lined stainless steel reactor and heated at 433 K for 3 d. On completion of the reaction, the reactor was cooled slowly to room temperature and the mixture was filtered, giving yellow blocky single crystals suitable for X-ray analysis in 38% yield.

### Refinement

All H atoms were positioned geometrically and refined using a riding model [C—H = 0.93 Å and O—H = 0.82 Å, *U*_iso_(H) = 1.2*U*_eq_(C,*O*)].

### Crystal data

**Table:**

[Cd(C_9_H_7_O_3_)_2_(C_10_H_8_N_2_)_2_]	*F*(000) = 1528
*M**_r_* = 751.06	*D*_x_ = 1.458 Mg m^−^^3^
Monoclinic, *P*2_1_/*c*	Mo *K*α radiation, λ = 0.71073 Å
Hall symbol: -P 2ybc	Cell parameters from 9455 reflections
*a* = 8.7769 (16) Å	θ = 2.0–27.6°
*b* = 36.711 (7) Å	µ = 0.69 mm^−^^1^
*c* = 13.188 (2) Å	*T* = 296 K
β = 126.382 (10)°	Block, yellow
*V* = 3421.0 (10) Å^3^	0.35 × 0.25 × 0.10 mm
*Z* = 4	

### Data collection

**Table:**

Bruker APEXII CCD area-detector diffractometer	7869 independent reflections
Radiation source: fine-focus sealed tube	6136 reflections with *I* > 2σ(*I*)
graphite	*R*_int_ = 0.034
φ and ω scans	θ_max_ = 27.6°, θ_min_ = 2.0°
Absorption correction: multi-scan (*SADABS*; Bruker, 2009)	*h* = −11→11
*T*_min_ = 0.81, *T*_max_ = 0.93	*k* = −45→47
29901 measured reflections	*l* = −12→17

### Refinement

**Table:**

Refinement on *F*^2^	Primary atom site location: structure-invariant direct methods
Least-squares matrix: full	Secondary atom site location: difference Fourier map
*R*[*F*^2^ > 2σ(*F*^2^)] = 0.036	Hydrogen site location: inferred from neighbouring sites
*wR*(*F*^2^) = 0.076	H-atom parameters constrained
*S* = 1.02	*w* = 1/[σ^2^(*F*_o_^2^) + (0.0265*P*)^2^ + 1.7042*P*] where *P* = (*F*_o_^2^ + 2*F*_c_^2^)/3
7869 reflections	(Δ/σ)_max_ = 0.002
442 parameters	Δρ_max_ = 0.48 e Å^−^^3^
1 restraint	Δρ_min_ = −0.53 e Å^−^^3^

### Special details

**Table:**

Geometry. All s.u.'s (except the s.u. in the dihedral angle between two l.s. planes) are estimated using the full covariance matrix. The cell s.u.'s are taken into account individually in the estimation of s.u.'s in distances, angles and torsion angles; correlations between s.u.'s in cell parameters are only used when they are defined by crystal symmetry. An approximate (isotropic) treatment of cell s.u.'s is used for estimating s.u.'s involving l.s. planes.
Refinement. Refinement of *F*^2^ against ALL reflections. The weighted *R*-factor *wR* and goodness of fit *S* are based on *F*^2^, conventional *R*-factors *R* are based on *F*, with *F* set to zero for negative *F*^2^. The threshold expression of *F*^2^ > σ(*F*^2^) is used only for calculating *R*-factors(gt) *etc*. and is not relevant to the choice of reflections for refinement. *R*-factors based on *F*^2^ are statistically about twice as large as those based on *F*, and *R*- factors based on ALL data will be even larger.

### Fractional atomic coordinates and isotropic or equivalent isotropic displacement parameters (Å^2^)

**Table:**

	*x*	*y*	*z*	*U*_iso_*/*U*_eq_	
Cd1	−0.04645 (2)	0.375575 (5)	−0.026646 (16)	0.03575 (6)	
C1	−0.4528 (4)	0.43853 (9)	0.4179 (3)	0.0566 (7)	
C2	−0.3126 (4)	0.45796 (10)	0.5215 (3)	0.0659 (9)	
H2A	−0.3356	0.4682	0.5758	0.079*	
C3	−0.1399 (4)	0.46216 (9)	0.5447 (3)	0.0634 (8)	
H3A	−0.0455	0.4752	0.6150	0.076*	
C4	−0.1046 (4)	0.44724 (8)	0.4651 (2)	0.0520 (7)	
H4A	0.0133	0.4504	0.4815	0.062*	
C5	−0.2440 (3)	0.42738 (7)	0.3599 (2)	0.0397 (6)	
C6	−0.4180 (4)	0.42272 (8)	0.3377 (2)	0.0467 (6)	
H6A	−0.5116	0.4090	0.2691	0.056*	
C7	−0.2141 (3)	0.41185 (7)	0.2705 (2)	0.0400 (6)	
H7A	−0.3146	0.3988	0.2032	0.048*	
C8	−0.0614 (4)	0.41434 (8)	0.2754 (2)	0.0483 (7)	
H8A	0.0442	0.4257	0.3448	0.058*	
C9	−0.0479 (4)	0.39991 (7)	0.1755 (3)	0.0450 (6)	
C10	1.0806 (3)	0.27718 (8)	0.3225 (3)	0.0497 (7)	
C11	1.1024 (4)	0.24959 (8)	0.4007 (3)	0.0543 (7)	
H11A	1.2113	0.2353	0.4431	0.065*	
C12	0.9631 (4)	0.24317 (8)	0.4159 (3)	0.0571 (8)	
H12A	0.9792	0.2249	0.4704	0.069*	
C13	0.8003 (4)	0.26347 (8)	0.3511 (3)	0.0519 (7)	
H13A	0.7063	0.2587	0.3616	0.062*	
C14	0.7738 (3)	0.29118 (7)	0.2697 (2)	0.0385 (5)	
C15	0.9169 (3)	0.29806 (7)	0.2571 (2)	0.0452 (6)	
H15A	0.9033	0.3167	0.2046	0.054*	
C16	0.6011 (3)	0.31352 (7)	0.1980 (2)	0.0385 (5)	
H16A	0.5979	0.3320	0.1485	0.046*	
C17	0.4518 (3)	0.31013 (7)	0.1967 (2)	0.0415 (6)	
H17A	0.4522	0.2921	0.2464	0.050*	
C18	0.2812 (3)	0.33360 (7)	0.1201 (2)	0.0403 (6)	
C19	0.0096 (5)	0.36621 (9)	−0.2536 (3)	0.0652 (9)	
H19A	0.1221	0.3780	−0.1933	0.078*	
C20	−0.0296 (6)	0.35961 (11)	−0.3692 (3)	0.0792 (10)	
H20A	0.0537	0.3669	−0.3871	0.095*	
C21	−0.1927 (6)	0.34225 (12)	−0.4564 (3)	0.0928 (13)	
H21A	−0.2237	0.3376	−0.5360	0.111*	
C22	−0.3119 (6)	0.33158 (11)	−0.4275 (3)	0.0853 (11)	
H22A	−0.4242	0.3195	−0.4869	0.102*	
C23	−0.2633 (4)	0.33893 (8)	−0.3082 (3)	0.0531 (7)	
C24	−0.3793 (4)	0.32705 (8)	−0.2669 (3)	0.0523 (7)	
C25	−0.5515 (5)	0.30908 (11)	−0.3469 (3)	0.0819 (11)	
H25A	−0.6010	0.3053	−0.4310	0.098*	
C26	−0.6470 (5)	0.29702 (12)	−0.3015 (4)	0.0934 (13)	
H26A	−0.7619	0.2850	−0.3543	0.112*	
C27	−0.5740 (5)	0.30267 (10)	−0.1791 (4)	0.0812 (11)	
H27A	−0.6368	0.2944	−0.1463	0.097*	
C28	−0.4037 (4)	0.32097 (8)	−0.1041 (3)	0.0607 (8)	
H28A	−0.3535	0.3250	−0.0201	0.073*	
C29	−0.4742 (4)	0.41556 (9)	−0.1822 (3)	0.0717 (10)	
H29A	−0.5005	0.3932	−0.1628	0.086*	
C30	−0.6181 (4)	0.44032 (10)	−0.2474 (3)	0.0723 (9)	
H30A	−0.7392	0.4348	−0.2722	0.087*	
C31	−0.5795 (5)	0.47320 (10)	−0.2748 (3)	0.0736 (10)	
H31A	−0.6745	0.4906	−0.3192	0.088*	
C32	−0.4000 (4)	0.48052 (9)	−0.2365 (3)	0.0641 (8)	
H32A	−0.3714	0.5029	−0.2544	0.077*	
C33	−0.2614 (4)	0.45420 (7)	−0.1708 (2)	0.0451 (6)	
C34	−0.0622 (4)	0.46075 (7)	−0.1234 (2)	0.0425 (6)	
C35	−0.0035 (5)	0.49414 (8)	−0.1388 (3)	0.0604 (8)	
H35A	−0.0897	0.5130	−0.1817	0.072*	
C36	0.1825 (5)	0.49878 (9)	−0.0899 (3)	0.0710 (9)	
H36A	0.2249	0.5209	−0.0990	0.085*	
C37	0.3057 (5)	0.47050 (9)	−0.0276 (3)	0.0685 (9)	
H37A	0.4331	0.4732	0.0066	0.082*	
C38	0.2389 (4)	0.43809 (8)	−0.0160 (3)	0.0551 (7)	
H38A	0.3232	0.4189	0.0259	0.066*	
N1	−0.1041 (3)	0.35667 (7)	−0.2231 (2)	0.0512 (6)	
N2	−0.3087 (3)	0.33299 (6)	−0.1467 (2)	0.0464 (5)	
N3	−0.2993 (3)	0.42174 (6)	−0.1454 (2)	0.0531 (6)	
N4	0.0578 (3)	0.43312 (6)	−0.06265 (19)	0.0434 (5)	
O1	0.0944 (3)	0.40896 (5)	0.18017 (18)	0.0572 (5)	
O2	−0.1759 (3)	0.38054 (5)	0.09022 (18)	0.0535 (5)	
O3	−0.6236 (3)	0.43639 (8)	0.3960 (2)	0.0870 (8)	
H3B	−0.7004	0.4274	0.3263	0.131*	
O4	0.2746 (3)	0.35843 (5)	0.05366 (18)	0.0545 (5)	
O5	0.1439 (2)	0.32674 (5)	0.12435 (17)	0.0486 (4)	
O6	1.2247 (3)	0.28348 (7)	0.3133 (2)	0.0813 (7)	
H6B	1.1878	0.2969	0.2532	0.122*	

### Atomic displacement parameters (Å^2^)

**Table:**

	*U*^11^	*U*^22^	*U*^33^	*U*^12^	*U*^13^	*U*^23^
Cd1	0.02886 (9)	0.04075 (11)	0.03889 (10)	0.00123 (7)	0.02077 (8)	0.00029 (8)
C1	0.0433 (15)	0.083 (2)	0.0555 (17)	0.0052 (14)	0.0358 (15)	−0.0005 (16)
C2	0.0562 (18)	0.104 (3)	0.0470 (17)	0.0076 (17)	0.0356 (16)	−0.0120 (17)
C3	0.0442 (16)	0.095 (2)	0.0450 (16)	0.0007 (15)	0.0234 (14)	−0.0170 (16)
C4	0.0375 (14)	0.072 (2)	0.0477 (16)	0.0001 (13)	0.0260 (13)	−0.0068 (14)
C5	0.0353 (12)	0.0474 (15)	0.0408 (13)	0.0031 (11)	0.0249 (11)	0.0019 (11)
C6	0.0395 (14)	0.0571 (17)	0.0468 (15)	−0.0007 (12)	0.0275 (13)	−0.0012 (13)
C7	0.0377 (13)	0.0425 (15)	0.0428 (14)	0.0013 (10)	0.0255 (12)	−0.0021 (11)
C8	0.0436 (14)	0.0612 (18)	0.0492 (16)	−0.0062 (12)	0.0325 (13)	−0.0122 (13)
C9	0.0496 (15)	0.0486 (16)	0.0501 (16)	0.0069 (12)	0.0368 (14)	0.0027 (13)
C10	0.0352 (13)	0.0610 (18)	0.0561 (17)	0.0083 (12)	0.0287 (13)	0.0068 (14)
C11	0.0389 (14)	0.0587 (18)	0.0586 (17)	0.0178 (13)	0.0252 (14)	0.0140 (14)
C12	0.0479 (16)	0.0545 (18)	0.0661 (19)	0.0091 (13)	0.0322 (15)	0.0222 (15)
C13	0.0375 (14)	0.0545 (17)	0.0653 (18)	0.0040 (12)	0.0313 (14)	0.0167 (14)
C14	0.0292 (11)	0.0413 (14)	0.0440 (14)	0.0030 (10)	0.0212 (11)	0.0036 (11)
C15	0.0366 (13)	0.0522 (16)	0.0479 (15)	0.0069 (11)	0.0258 (12)	0.0111 (12)
C16	0.0344 (12)	0.0401 (14)	0.0411 (13)	0.0051 (10)	0.0224 (11)	0.0061 (11)
C17	0.0331 (12)	0.0414 (14)	0.0505 (15)	0.0057 (10)	0.0251 (12)	0.0094 (12)
C18	0.0291 (12)	0.0440 (15)	0.0415 (14)	0.0031 (10)	0.0176 (11)	−0.0043 (12)
C19	0.072 (2)	0.076 (2)	0.065 (2)	−0.0132 (16)	0.0500 (19)	−0.0133 (16)
C20	0.106 (3)	0.085 (3)	0.079 (3)	−0.010 (2)	0.073 (3)	−0.008 (2)
C21	0.120 (3)	0.114 (3)	0.062 (2)	−0.019 (3)	0.063 (3)	−0.021 (2)
C22	0.090 (3)	0.106 (3)	0.055 (2)	−0.023 (2)	0.040 (2)	−0.027 (2)
C23	0.0509 (16)	0.0584 (18)	0.0416 (15)	−0.0007 (13)	0.0229 (14)	−0.0064 (13)
C24	0.0435 (15)	0.0516 (17)	0.0480 (16)	−0.0036 (12)	0.0197 (13)	−0.0086 (13)
C25	0.060 (2)	0.104 (3)	0.064 (2)	−0.028 (2)	0.0279 (18)	−0.031 (2)
C26	0.061 (2)	0.110 (3)	0.096 (3)	−0.037 (2)	0.039 (2)	−0.036 (3)
C27	0.066 (2)	0.087 (3)	0.103 (3)	−0.0295 (19)	0.057 (2)	−0.020 (2)
C28	0.0609 (18)	0.061 (2)	0.067 (2)	−0.0172 (15)	0.0423 (17)	−0.0117 (16)
C29	0.0447 (17)	0.063 (2)	0.100 (3)	0.0120 (14)	0.0385 (18)	0.0190 (18)
C30	0.0460 (17)	0.083 (3)	0.078 (2)	0.0140 (16)	0.0317 (17)	0.008 (2)
C31	0.062 (2)	0.078 (2)	0.076 (2)	0.0325 (18)	0.0380 (19)	0.0200 (19)
C32	0.066 (2)	0.057 (2)	0.072 (2)	0.0205 (15)	0.0425 (18)	0.0197 (16)
C33	0.0514 (15)	0.0463 (16)	0.0385 (14)	0.0114 (12)	0.0272 (13)	0.0037 (12)
C34	0.0542 (15)	0.0411 (15)	0.0381 (14)	0.0002 (12)	0.0306 (13)	−0.0020 (11)
C35	0.071 (2)	0.0463 (18)	0.065 (2)	0.0025 (15)	0.0411 (18)	0.0054 (15)
C36	0.080 (2)	0.051 (2)	0.082 (2)	−0.0148 (16)	0.048 (2)	0.0049 (17)
C37	0.0551 (18)	0.066 (2)	0.080 (2)	−0.0126 (16)	0.0374 (18)	0.0048 (18)
C38	0.0440 (15)	0.0523 (17)	0.0607 (18)	−0.0062 (13)	0.0265 (14)	0.0035 (14)
N1	0.0540 (14)	0.0567 (15)	0.0470 (13)	−0.0049 (11)	0.0322 (12)	−0.0078 (11)
N2	0.0427 (12)	0.0454 (13)	0.0491 (13)	−0.0056 (10)	0.0261 (11)	−0.0051 (10)
N3	0.0416 (12)	0.0477 (14)	0.0673 (15)	0.0094 (10)	0.0309 (12)	0.0105 (12)
N4	0.0428 (12)	0.0427 (13)	0.0439 (12)	−0.0009 (9)	0.0254 (10)	0.0010 (10)
O1	0.0630 (12)	0.0674 (13)	0.0661 (11)	−0.0076 (10)	0.0519 (11)	−0.0153 (9)
O2	0.0521 (11)	0.0628 (13)	0.0497 (11)	0.0022 (9)	0.0324 (10)	−0.0086 (10)
O3	0.0528 (13)	0.147 (2)	0.0842 (16)	−0.0142 (14)	0.0532 (13)	−0.0328 (16)
O4	0.0469 (11)	0.0551 (12)	0.0604 (12)	0.0145 (9)	0.0312 (10)	0.0181 (10)
O5	0.0300 (9)	0.0545 (11)	0.0605 (11)	0.0048 (8)	0.0264 (9)	−0.0008 (9)
O6	0.0448 (11)	0.120 (2)	0.0959 (17)	0.0310 (12)	0.0506 (13)	0.0445 (15)

### Geometric parameters (Å, °)

**Table:**

Cd1—O2	2.4033 (18)	C19—N1	1.325 (4)
Cd1—N1	2.428 (2)	C19—C20	1.371 (4)
Cd1—O4	2.4325 (18)	C19—H19A	0.9300
Cd1—N2	2.434 (2)	C20—C21	1.351 (5)
Cd1—O5	2.4544 (18)	C20—H20A	0.9300
Cd1—N4	2.458 (2)	C21—C22	1.366 (5)
Cd1—N3	2.475 (2)	C21—H21A	0.9300
Cd1—O1	2.5435 (19)	C22—C23	1.391 (4)
C1—O3	1.351 (3)	C22—H22A	0.9300
C1—C2	1.377 (4)	C23—N1	1.332 (3)
C1—C6	1.390 (4)	C23—C24	1.477 (4)
C2—C3	1.366 (4)	C24—N2	1.334 (3)
C2—H2A	0.9300	C24—C25	1.393 (4)
C3—C4	1.373 (4)	C25—C26	1.361 (5)
C3—H3A	0.9300	C25—H25A	0.9300
C4—C5	1.393 (4)	C26—C27	1.353 (5)
C4—H4A	0.9300	C26—H26A	0.9300
C5—C6	1.385 (3)	C27—C28	1.382 (4)
C5—C7	1.466 (3)	C27—H27A	0.9300
C6—H6A	0.9300	C28—N2	1.327 (3)
C7—C8	1.306 (3)	C28—H28A	0.9300
C7—H7A	0.9300	C29—N3	1.327 (3)
C8—C9	1.488 (3)	C29—C30	1.368 (4)
C8—H8A	0.9300	C29—H29A	0.9300
C9—O2	1.239 (3)	C30—C31	1.359 (5)
C9—O1	1.259 (3)	C30—H30A	0.9300
C10—O6	1.360 (3)	C31—C32	1.366 (4)
C10—C11	1.376 (4)	C31—H31A	0.9300
C10—C15	1.388 (3)	C32—C33	1.384 (4)
C11—C12	1.372 (4)	C32—H32A	0.9300
C11—H11A	0.9300	C33—N3	1.333 (3)
C12—C13	1.371 (4)	C33—C34	1.486 (4)
C12—H12A	0.9300	C34—N4	1.334 (3)
C13—C14	1.395 (3)	C34—C35	1.390 (4)
C13—H13A	0.9300	C35—C36	1.364 (4)
C14—C15	1.385 (3)	C35—H35A	0.9300
C14—C16	1.471 (3)	C36—C37	1.367 (4)
C15—H15A	0.9300	C36—H36A	0.9300
C16—C17	1.307 (3)	C37—C38	1.374 (4)
C16—H16A	0.9300	C37—H37A	0.9300
C17—C18	1.487 (3)	C38—N4	1.334 (3)
C17—H17A	0.9300	C38—H38A	0.9300
C18—O4	1.241 (3)	O3—H3B	0.8200
C18—O5	1.264 (3)	O6—H6B	0.8200
			
O2—Cd1—N1	145.83 (7)	O4—C18—C17	120.8 (2)
O2—Cd1—O4	127.34 (7)	O5—C18—C17	117.1 (2)
N1—Cd1—O4	81.09 (7)	N1—C19—C20	123.6 (3)
O2—Cd1—N2	80.88 (7)	N1—C19—H19A	118.2
N1—Cd1—N2	66.68 (8)	C20—C19—H19A	118.2
O4—Cd1—N2	121.41 (7)	C21—C20—C19	118.1 (3)
O2—Cd1—O5	83.59 (6)	C21—C20—H20A	121.0
N1—Cd1—O5	105.53 (7)	C19—C20—H20A	121.0
O4—Cd1—O5	53.31 (6)	C20—C21—C22	119.9 (3)
N2—Cd1—O5	89.12 (7)	C20—C21—H21A	120.1
O2—Cd1—N4	115.50 (7)	C22—C21—H21A	120.1
N1—Cd1—N4	84.57 (7)	C21—C22—C23	119.2 (3)
O4—Cd1—N4	80.71 (7)	C21—C22—H22A	120.4
N2—Cd1—N4	138.00 (7)	C23—C22—H22A	120.4
O5—Cd1—N4	129.22 (6)	N1—C23—C22	120.8 (3)
O2—Cd1—N3	76.48 (7)	N1—C23—C24	116.2 (2)
N1—Cd1—N3	88.83 (8)	C22—C23—C24	123.0 (3)
O4—Cd1—N3	145.59 (7)	N2—C24—C25	120.6 (3)
N2—Cd1—N3	83.18 (8)	N2—C24—C23	117.0 (2)
O5—Cd1—N3	159.52 (7)	C25—C24—C23	122.4 (3)
N4—Cd1—N3	65.55 (7)	C26—C25—C24	119.7 (3)
O2—Cd1—O1	52.40 (6)	C26—C25—H25A	120.2
N1—Cd1—O1	160.57 (7)	C24—C25—H25A	120.2
O4—Cd1—O1	87.53 (7)	C27—C26—C25	119.7 (3)
N2—Cd1—O1	132.66 (7)	C27—C26—H26A	120.1
O5—Cd1—O1	79.51 (6)	C25—C26—H26A	120.1
N4—Cd1—O1	78.06 (7)	C26—C27—C28	118.3 (3)
N3—Cd1—O1	91.81 (8)	C26—C27—H27A	120.8
O3—C1—C2	117.8 (3)	C28—C27—H27A	120.8
O3—C1—C6	122.0 (3)	N2—C28—C27	122.9 (3)
C2—C1—C6	120.1 (2)	N2—C28—H28A	118.6
C3—C2—C1	120.0 (3)	C27—C28—H28A	118.6
C3—C2—H2A	120.0	N3—C29—C30	123.6 (3)
C1—C2—H2A	120.0	N3—C29—H29A	118.2
C2—C3—C4	120.5 (3)	C30—C29—H29A	118.2
C2—C3—H3A	119.7	C31—C30—C29	118.3 (3)
C4—C3—H3A	119.7	C31—C30—H30A	120.8
C3—C4—C5	120.5 (3)	C29—C30—H30A	120.8
C3—C4—H4A	119.8	C30—C31—C32	119.4 (3)
C5—C4—H4A	119.8	C30—C31—H31A	120.3
C6—C5—C4	118.8 (2)	C32—C31—H31A	120.3
C6—C5—C7	118.9 (2)	C31—C32—C33	119.2 (3)
C4—C5—C7	122.3 (2)	C31—C32—H32A	120.4
C5—C6—C1	120.0 (3)	C33—C32—H32A	120.4
C5—C6—H6A	120.0	N3—C33—C32	121.6 (3)
C1—C6—H6A	120.0	N3—C33—C34	116.5 (2)
C8—C7—C5	126.9 (2)	C32—C33—C34	121.9 (3)
C8—C7—H7A	116.5	N4—C34—C35	121.9 (3)
C5—C7—H7A	116.5	N4—C34—C33	116.3 (2)
C7—C8—C9	123.4 (2)	C35—C34—C33	121.8 (2)
C7—C8—H8A	118.3	C36—C35—C34	119.0 (3)
C9—C8—H8A	118.3	C36—C35—H35A	120.5
O2—C9—O1	122.3 (2)	C34—C35—H35A	120.5
O2—C9—C8	120.2 (2)	C35—C36—C37	119.2 (3)
O1—C9—C8	117.5 (2)	C35—C36—H36A	120.4
O6—C10—C11	117.9 (2)	C37—C36—H36A	120.4
O6—C10—C15	121.7 (3)	C36—C37—C38	119.1 (3)
C11—C10—C15	120.3 (2)	C36—C37—H37A	120.4
C12—C11—C10	119.8 (2)	C38—C37—H37A	120.4
C12—C11—H11A	120.1	N4—C38—C37	122.5 (3)
C10—C11—H11A	120.1	N4—C38—H38A	118.7
C13—C12—C11	120.4 (3)	C37—C38—H38A	118.7
C13—C12—H12A	119.8	C19—N1—C23	118.4 (2)
C11—C12—H12A	119.8	C19—N1—Cd1	122.03 (19)
C12—C13—C14	120.8 (2)	C23—N1—Cd1	119.27 (18)
C12—C13—H13A	119.6	C28—N2—C24	118.9 (2)
C14—C13—H13A	119.6	C28—N2—Cd1	121.84 (18)
C15—C14—C13	118.5 (2)	C24—N2—Cd1	118.14 (18)
C15—C14—C16	118.9 (2)	C29—N3—C33	117.9 (2)
C13—C14—C16	122.6 (2)	C29—N3—Cd1	121.6 (2)
C14—C15—C10	120.2 (2)	C33—N3—Cd1	120.49 (17)
C14—C15—H15A	119.9	C38—N4—C34	118.3 (2)
C10—C15—H15A	119.9	C38—N4—Cd1	120.46 (18)
C17—C16—C14	126.8 (2)	C34—N4—Cd1	121.18 (16)
C17—C16—H16A	116.6	C9—O1—Cd1	89.06 (16)
C14—C16—H16A	116.6	C9—O2—Cd1	96.16 (16)
C16—C17—C18	123.5 (2)	C1—O3—H3B	109.5
C16—C17—H17A	118.3	C18—O4—Cd1	93.11 (15)
C18—C17—H17A	118.3	C18—O5—Cd1	91.51 (15)
O4—C18—O5	122.1 (2)	C10—O6—H6B	109.5
			
O3—C1—C2—C3	−177.3 (3)	O5—Cd1—N2—C28	70.6 (2)
C6—C1—C2—C3	0.9 (5)	N4—Cd1—N2—C28	−131.3 (2)
C1—C2—C3—C4	0.2 (5)	N3—Cd1—N2—C28	−90.4 (2)
C2—C3—C4—C5	−0.4 (5)	O1—Cd1—N2—C28	−4.2 (3)
C3—C4—C5—C6	−0.5 (4)	O2—Cd1—N2—C24	154.5 (2)
C3—C4—C5—C7	178.3 (3)	N1—Cd1—N2—C24	−14.47 (19)
C4—C5—C6—C1	1.7 (4)	O4—Cd1—N2—C24	−77.0 (2)
C7—C5—C6—C1	−177.2 (3)	O5—Cd1—N2—C24	−121.8 (2)
O3—C1—C6—C5	176.3 (3)	N4—Cd1—N2—C24	36.3 (2)
C2—C1—C6—C5	−1.9 (5)	N3—Cd1—N2—C24	77.2 (2)
C6—C5—C7—C8	178.4 (3)	O1—Cd1—N2—C24	163.36 (18)
C4—C5—C7—C8	−0.4 (4)	C30—C29—N3—C33	1.6 (5)
C5—C7—C8—C9	−175.5 (2)	C30—C29—N3—Cd1	179.6 (3)
C7—C8—C9—O2	−9.4 (4)	C32—C33—N3—C29	−1.7 (4)
C7—C8—C9—O1	168.8 (3)	C34—C33—N3—C29	177.5 (3)
O6—C10—C11—C12	177.8 (3)	C32—C33—N3—Cd1	−179.7 (2)
C15—C10—C11—C12	−1.2 (5)	C34—C33—N3—Cd1	−0.5 (3)
C10—C11—C12—C13	1.7 (5)	O2—Cd1—N3—C29	−51.6 (3)
C11—C12—C13—C14	−0.6 (5)	N1—Cd1—N3—C29	97.2 (3)
C12—C13—C14—C15	−0.9 (4)	O4—Cd1—N3—C29	169.5 (2)
C12—C13—C14—C16	179.8 (3)	N2—Cd1—N3—C29	30.6 (3)
C13—C14—C15—C10	1.3 (4)	O5—Cd1—N3—C29	−38.0 (4)
C16—C14—C15—C10	−179.3 (2)	N4—Cd1—N3—C29	−178.2 (3)
O6—C10—C15—C14	−179.2 (3)	O1—Cd1—N3—C29	−102.2 (3)
C11—C10—C15—C14	−0.3 (4)	O2—Cd1—N3—C33	126.3 (2)
C15—C14—C16—C17	178.3 (3)	N1—Cd1—N3—C33	−84.8 (2)
C13—C14—C16—C17	−2.4 (4)	O4—Cd1—N3—C33	−12.5 (3)
C14—C16—C17—C18	−179.2 (2)	N2—Cd1—N3—C33	−151.5 (2)
C16—C17—C18—O4	−1.1 (4)	O5—Cd1—N3—C33	139.9 (2)
C16—C17—C18—O5	177.9 (2)	N4—Cd1—N3—C33	−0.25 (19)
N1—C19—C20—C21	−0.4 (6)	O1—Cd1—N3—C33	75.8 (2)
C19—C20—C21—C22	−0.6 (6)	C37—C38—N4—C34	0.5 (4)
C20—C21—C22—C23	0.2 (7)	C37—C38—N4—Cd1	−176.2 (2)
C21—C22—C23—N1	1.1 (6)	C35—C34—N4—C38	−0.1 (4)
C21—C22—C23—C24	−177.3 (3)	C33—C34—N4—C38	−178.5 (2)
N1—C23—C24—N2	−4.2 (4)	C35—C34—N4—Cd1	176.5 (2)
C22—C23—C24—N2	174.2 (3)	C33—C34—N4—Cd1	−1.8 (3)
N1—C23—C24—C25	178.3 (3)	O2—Cd1—N4—C38	117.8 (2)
C22—C23—C24—C25	−3.3 (5)	N1—Cd1—N4—C38	−91.1 (2)
N2—C24—C25—C26	−1.0 (6)	O4—Cd1—N4—C38	−9.2 (2)
C23—C24—C25—C26	176.4 (3)	N2—Cd1—N4—C38	−136.7 (2)
C24—C25—C26—C27	0.1 (6)	O5—Cd1—N4—C38	14.6 (2)
C25—C26—C27—C28	0.5 (6)	N3—Cd1—N4—C38	177.8 (2)
C26—C27—C28—N2	−0.4 (6)	O1—Cd1—N4—C38	80.2 (2)
N3—C29—C30—C31	−0.7 (6)	O2—Cd1—N4—C34	−58.8 (2)
C29—C30—C31—C32	−0.2 (5)	N1—Cd1—N4—C34	92.33 (19)
C30—C31—C32—C33	0.1 (5)	O4—Cd1—N4—C34	174.16 (19)
C31—C32—C33—N3	0.9 (5)	N2—Cd1—N4—C34	46.7 (2)
C31—C32—C33—C34	−178.3 (3)	O5—Cd1—N4—C34	−162.03 (16)
N3—C33—C34—N4	1.5 (3)	N3—Cd1—N4—C34	1.15 (17)
C32—C33—C34—N4	−179.3 (2)	O1—Cd1—N4—C34	−96.41 (19)
N3—C33—C34—C35	−176.9 (3)	O2—C9—O1—Cd1	2.4 (3)
C32—C33—C34—C35	2.3 (4)	C8—C9—O1—Cd1	−175.8 (2)
N4—C34—C35—C36	−0.2 (4)	O2—Cd1—O1—C9	−1.33 (15)
C33—C34—C35—C36	178.1 (3)	N1—Cd1—O1—C9	161.6 (2)
C34—C35—C36—C37	0.1 (5)	O4—Cd1—O1—C9	−144.40 (16)
C35—C36—C37—C38	0.2 (5)	N2—Cd1—O1—C9	−12.4 (2)
C36—C37—C38—N4	−0.5 (5)	O5—Cd1—O1—C9	−91.29 (16)
C20—C19—N1—C23	1.6 (5)	N4—Cd1—O1—C9	134.57 (17)
C20—C19—N1—Cd1	−172.3 (3)	N3—Cd1—O1—C9	70.03 (16)
C22—C23—N1—C19	−1.9 (5)	O1—C9—O2—Cd1	−2.6 (3)
C24—C23—N1—C19	176.5 (3)	C8—C9—O2—Cd1	175.6 (2)
C22—C23—N1—Cd1	172.2 (3)	N1—Cd1—O2—C9	−168.65 (16)
C24—C23—N1—Cd1	−9.4 (3)	O4—Cd1—O2—C9	50.39 (19)
O2—Cd1—N1—C19	166.6 (2)	N2—Cd1—O2—C9	173.17 (17)
O4—Cd1—N1—C19	−43.9 (2)	O5—Cd1—O2—C9	83.04 (16)
N2—Cd1—N1—C19	−173.8 (3)	N4—Cd1—O2—C9	−47.60 (18)
O5—Cd1—N1—C19	−91.7 (2)	N3—Cd1—O2—C9	−101.71 (17)
N4—Cd1—N1—C19	37.5 (2)	O1—Cd1—O2—C9	1.36 (15)
N3—Cd1—N1—C19	103.1 (2)	O5—C18—O4—Cd1	−0.5 (3)
O1—Cd1—N1—C19	11.0 (4)	C17—C18—O4—Cd1	178.4 (2)
O2—Cd1—N1—C23	−7.3 (3)	O2—Cd1—O4—C18	42.24 (18)
O4—Cd1—N1—C23	142.2 (2)	N1—Cd1—O4—C18	−116.77 (17)
N2—Cd1—N1—C23	12.3 (2)	N2—Cd1—O4—C18	−61.19 (17)
O5—Cd1—N1—C23	94.4 (2)	O5—Cd1—O4—C18	0.28 (14)
N4—Cd1—N1—C23	−136.3 (2)	N4—Cd1—O4—C18	157.32 (16)
N3—Cd1—N1—C23	−70.8 (2)	N3—Cd1—O4—C18	168.62 (15)
O1—Cd1—N1—C23	−162.9 (2)	O1—Cd1—O4—C18	79.02 (16)
C27—C28—N2—C24	−0.4 (5)	O4—C18—O5—Cd1	0.5 (3)
C27—C28—N2—Cd1	167.1 (3)	C17—C18—O5—Cd1	−178.4 (2)
C25—C24—N2—C28	1.1 (4)	O2—Cd1—O5—C18	−147.93 (15)
C23—C24—N2—C28	−176.4 (3)	N1—Cd1—O5—C18	65.67 (15)
C25—C24—N2—Cd1	−166.8 (3)	O4—Cd1—O5—C18	−0.27 (14)
C23—C24—N2—Cd1	15.6 (3)	N2—Cd1—O5—C18	131.15 (15)
O2—Cd1—N2—C28	−13.0 (2)	N4—Cd1—O5—C18	−30.07 (17)
N1—Cd1—N2—C28	178.0 (2)	N3—Cd1—O5—C18	−161.2 (2)
O4—Cd1—N2—C28	115.4 (2)	O1—Cd1—O5—C18	−95.06 (15)

### Hydrogen-bond geometry (Å, °)

**Table:**

*D*—H···*A*	*D*—H	H···*A*	*D*···*A*	*D*—H···*A*
O6—H6B···O5^i^	0.82	1.86	2.672 (3)	171.
O3—H3B···O1^ii^	0.82	1.82	2.624 (3)	168.

Symmetry codes: (i) *x*+1, *y*, *z*; (ii) *x*−1, *y*, *z*.
